# Reusable Personal Protective Equipment Viewed Through the Lens of Sustainability

**DOI:** 10.1016/j.identj.2024.07.1270

**Published:** 2024-11-06

**Authors:** Laurence J. Walsh

**Affiliations:** School of Dentistry, The University of Queensland, Brisbane, Australia

**Keywords:** PPE, Sustainability, Masks, Respirators, Gowns, Environmental impact

## Abstract

From early 2020 the COVID-19 pandemic drove dramatic increases in the production and use of single use disposable masks, respirators and gowns, and highlighted the vulnerability of supply chains for these items. This paper explores the impacts of the rising demands for these single use items through the lens of sustainability, by collating data on the carbon footprint and other impacts, and then discussing challenges, solutions, and future perspectives. Polypropylene and other key synthetic fibre components of these items are not biodegradable, and persist in the environments for prolonged periods generating microplastics as they degrade slowly. Various methods have been shown to allow limited repeated use of surgical masks and respirators, and this has spurred the development of masks and respirators designed for many cycles of reuse. Parallel discussions around gowns reveal that reuseable gowns offer many advantages for performance as well as reduced environmental impact. At the local dental clinic level, those making purchasing decisions should consider impacts of their product choices on the environment. Such impacts occur from manufacture, transport, and disposal of PPE, and from degradation within the environment. Regulators need to encourage use of reuseable items and facilitate this through local guidelines, while at the international level, more work is needed to develop uniform standards for reuseable masks, respirators and gowns.

## Introduction

Disposable polypropylene surgical masks and gowns date from the mid-1960s. Demands for these and other disposable items of personal protective equipment (PPE) in healthcare soared from early 2020 as the COVID-19 pandemic took hold, driven by concerns over the risks of cross contamination, and a perception of hygienic superiority for single-use items over reusable alternatives.^1^

During 2020, wearing single use PPE became the norm for dental staff and other healthcare workers in many countries around the world, driven by government and regulatory directives. By March 2020, the global need for surgical masks in healthcare settings had risen to around 90 million per month, according to estimates from the World Health Organisation. This was before widespread mandates were introduced around mask wearing in the community.^2^ Future projections are that in the years following 2023 (after the end of the emergency declaration of the pandemic), there will be compound annual growth for surgical masks by around 20% per annum until 2025.^3^ These impressive statistics emphasise the need to consider ways to contain the impacts of these items of PPE on the environment.

The production of polypropylene, polyester, and other synthetic fibres from oil is a large contributor to the component of total global carbon emissions associated from textile production. In light of these concerns, this article lays out some of the major directions which have been explored to meet the challenge of sustainability for reusable PPE in the healthcare setting, and identifies major topics where further research is needed.

## Environmental impacts of single use PPE

### Methods of manufacture

The predominant polymer used in surgical masks, respirators and gowns is polypropylene. In some cases, the primary mask material of polypropylene is combined with polyester or other synthetic polymers. In masks and respirators, the nose clip is typically made of polyvinylchloride alone or the same material covering a core of aluminium.^4^ The ear loops of single-use masks is typically polyetherimide, a material which generates in its production far more greenhouse gas emissions than synthetic rubber or polyester elastic straps.^5^

### Production

Polyethylene and other polymeric or plastic materials are derived from fossil fuels, and their production generates sizeable greenhouse gas emissions, at around 2.9 kg of carbon dioxide equivalent per kg of material.^5^ Spunbond-meltblown-spunbond (SMS) construction is used for the inner and outermost layers of masks and respirators, and also for the production of disposable gowns. The material is formed as a set of directly entangled textile fibres, which are bonded together to increase the overall strength. The bonding process can employ a range of methods including heat and hydro entanglement.^6^ The latter gives a soft feel to the material, and thus greater user comfort. Various surface modifications are then applied, such as polymer coatings to improve fluid resistance. SMS construction gives high breathability to the end product. Melt-blown extrusion is used to produce the very fine fibres in the inner filter layers of masks and respirators.

### Transport

The majority of surgical masks, respirators and gowns used globally are manufactured in Asia, and the polymers are mostly produced in China,^7^ making this country the manufacturing hub for the global market, including both finished items of disposable PPE and the raw materials needed to produce them elsewhere. Production of these 3 types of PPE within China was estimated to have increased by as much as tenfold during the second half of 2020, with production reaching 200 million masks per day.^2^ On the global scale, China represented 77% of 2020 exports for masks, respirators and gowns.^2^ As a consequence, shipping costs contribute to the total greenhouse gas emissions of these items of PPE.

On an individual item basis, disposable gowns have the greatest carbon footprint of the 3 (expressed as grams of carbon dioxide gas equivalents) at 905 g, followed by respirators (76-125 g), and then surgical masks (13-20 g). This reflects the higher costs of production when there is a larger quantity of materials involved.^8^

### Waste

It has been estimated that 3.5 million metric tons of surgical masks went into landfill in 2020 alone, and this rose to over 293,000 tons per month (3.5 million tonnes per year) in 2021 when the use of disposable surgical facemasks in healthcare and in the wider community reached almost 6 billion per day, of which around 2.4 billion masks ended up eventually in landfill.^9^ Some of this waste went into landfills and open dumps that were uncontrolled. Indiscriminate dumping has led to waste items of PPE spreading quickly into the environment,^10^ including into the ocean since polypropylene is less dense than seawater, and can spread along ocean currents.^11^ While they are floating, masks may take hundreds of years to degrade, depending on the ambient temperature and exposure to sunlight. They eventually break down into microplastics.^4^

Studies using a life cycle analysis (LCA) approach have shown that disposable surgical masks and disposable gowns have caused a surge in environmental impacts in terms of energy usage, water usage, greenhouse gas emissions, and solid waste generation.^10^^,^^12^ Greenhouse gas emissions arise from the coal that is used to produce energy. Emissions increase considerably when waste from the dental clinic is set for incineration rather than for landfill.^13^

What happens to used items of PPE varies by jurisdiction around the world, with some countries requiring incineration of all items classed as biomedical waste, while other countries use incineration for both domestic waste and biomedical waste, and combine this with ‘waste to energy’ technology.^1^ This created a challenge for incineration facilities when there was a dramatic rise in plastic waste production due to medical PPE use skyrocketing during the pandemic. Landfill of nonrecyclable plastics generates lower carbon dioxide emissions than incineration but suffers from many other challenges as a waste management approach.^1^

### Microplastics

As with surgical respirators and disposable gowns, single-use masks are predominantly made of polypropylene, a material that is not recyclable, degrades very slowly, and which can have detrimental impacts on the environment by generating microplastics.^14^^,^^15^ These microplastics can remain in the environment for hundreds of years, and affect the health of both humans and aquatic species, including entering the food chain for human consumption.^2^^,^^16^ Microplastics from PPE cause malnutrition in zooplankton and in other levels of the marine food chain, and so affect ocean productivity.^6^, ^7^, ^8^

### Carbon footprint

Attempts have been made to quantify the damage to human health, ecosystems, and resource scarcity from disposable PPE. As an example, in the United Kingdom in the first 6 months of the COVID-19 pandemic (25 February to 23 August 2020), the environmental impacts of items of PPE distributed to public sector health care workers has been estimated as a total carbon footprint of 106,478 tonnes of carbon dioxide equivalents. This equates to 591 tonnes per day, which is the same as around 244 return flights from London to New York.^6^ Put another way, the carbon footprint was 26,662 times an average person's carbon footprint during the same period. The estimated damage to human health was 239 disability adjusted life years, a loss of 0.47 species per year, and a financial impact from resource depletion of USD $12.7 million.

### Drivers for change

Overall, disposable surgical masks, respirators and gowns represent a significant component of waste from healthcare facilities,^3^ and this issue has been known for over 40 years.^17^ The ecological impacts of using disposable PPE arise from multiple points, including its manufacture, its transport to the dental clinic, and its disposal. This emphasises the need for alternatives such as items with extended use capability, reusable items of PPE, and the need to explore opportunities to recycle items, or to convert them (up cycle or down cycle).

Single-use disposable products manufactured overseas are prone to supply chain disruptions from global events. During 2020, interruptions in manufacturing and supply chain interruptions greatly affected the global supply of PPE. This led to considerable thinking around strategies to conserve existing PPE, and to extend the use times of PPE.^18^

The statistics on the volume of disposable PPE reinforce the need to decrease plastic waste generation and increase recycling. Having said that, there are many challenges around recycling polypropylene. At the current time, it is estimated that only 3% of polypropylene is recovered and recycled.^3^ This is mostly because healthcare waste is contaminated and poses a risk to individuals who are working in recycling because of potentially infectious agents being present.

## Methods for reprocessing surgical masks and respirators

Key considerations for the decontamination and reprocessing of surgical masks and respirators are that the chosen method must remove or inactivate infectious agents, not compromise the function of the item itself, be available and practical in healthcare settings, and minimise risks to operators of the decontamination equipment and end users alike. Not all available decontamination technologies are appropriate for all brands and types of surgical masks and respirators.^19^^,^^20^

### Gamma irradiation

Gamma sterilisation is widely used for healthcare products, and highly effective for PPE,^21^ but is not suitable for local deployment because of the inherent risks and challenges of ionising radiation, and the need to transport materials off-site.

### Ethylene trioxide

Ethylene trioxide has been used to reprocess surgical respirators, and does not affect their performance, however, this method is not widely available, and has significant concerns relating to health impacts of the sterilant.^22^ The required cycle time of 1 hour at 55 °C is much longer than would be required than if some other reprocessing methods were used.

### Moist heat

In research work undertaken before the COVID-19 pandemic, different protocols using moist heat had shown useful results for respirators that have been experimentally contaminated (eg, with human influenza viruses, bacteriophage MS2, or *Bacillus subtilis* spores), with little influence on submicron filtration performance or fit to the face for a single steam sterilisation cycle.^23^, ^24^, ^25^, ^26^

On May 21, 2020, the U.S. Food and Drug Administration issued an Emergency Use Authorization to the STERIS Corporation for the emergency use of a particular low temperature steam sterilisation cycle (65 °C for 30 minutes at a relative humidity of 100%) for decontaminating compatible N95 respirators for single-user reuse by healthcare personnel, for a maximum of 4 cycles. At the time, there were insufficient supplies of N95 respirators, and there were no FDA approved or cleared devices for decontaminating N95 respirators. The Emergency Use Authorization was based on demonstrated performance against Feline calicivirus as a viral challenge, and against *Mycobacterium smegmatis* or *Mycobacterium hassiacum*, which are non-pathogenic surrogates of *Mycobacterium tuberculosis*.^19^ The tested surgical respirators showed no degradation in the nosefoam or headbands after 3 cycles, and no deterioration in fit. In the same study, respirators were incompatible with standard steam sterilisation cycles (121 °C), and all attempts at using temperatures greater than 75 °C adversely affected filtration performance and respirator fit.

### Dry heat

Another option is the application of dry heat, at 70-77 °C for 30 minutes, rather than the usual temperature of 160 °C for 2 hours, which will cause degradation.^27^^,^^28^ Similar issues arise with microwave treatment, where high temperatures cause polypropylene fibres to melt.^20^ A concern with repeated application of dry heat is degradation of fit, especially for respirators, due to heat induced changes in the respirator material. This could lead to loss of the hermetic seal against the skin of the face, allowing the entry of aerosols. One must also consider that degradation of elastic straps will occur with repeated exposure to dry heat.

### Vaporised hydrogen peroxide

Vaporised hydrogen peroxide (VHP) has emerged as a popular method for decontaminating masks and respirators, whilst maintaining their protective functions. It is highly effective at inactivating very large amounts of both SARS-CoV-2 and the common surrogate coronavirus HCoV-229E, and is compatible with common surgical respirator types (eg, 3M 8210 and 3M 9210+).^29^ A major advantage of VHP sterilisation is that it does not subject items in the load to high temperatures, as the typical maximum chamber temperature will be no more than 45 °C.^30^

In some studies, VHP sterilisation did not adversely affect elastic headbands or metallic inserts in masks or respirators,^19^^,^^31^ while in other work, prolonged usage of respirators followed by several cycles of VHP decontamination led to fit test failures in some brands.^32^ This reinforces the value of having objective data on individual products to inform users of how well suited the particular product in use is to undergo multiple cycles of decontamination using VHP.^33^, ^34^, ^35^

With multiple rounds of VHP decontamination, the fit of surgical respirators may decline, impairing the quality of their seal against the facial skin, and causing the loss of the airtight seal required for a respirator. Some studies have suggested that this may occur after 6 or 8 VHP cycles, depending on the brand of respirator that is used, and when there is a change from one user to another.^31^

Masks or respirators which contain significant amounts of sponge like foam or other porous materials around the bridge of the nose will be problematic during VHP sterilisation because hydrogen peroxide vapour may condense into liquid form in these areas, causing a drop in the concentration of sterilant within the chamber.^36^ This is one reason why the overall suitability of the mask or respirator needs to be assessed before considering using VHP sterilisation.

Another important point is that very old respirators which are past their recommended use date (i.e. expired) are unsuitable for reprocessing using VHP as they may have already failed to meet particle filtration requirements, because of degradation over long periods of time.^29^

### Germicidal ultraviolet C irradiation

Germicidal irradiation using ultraviolet C (UVC) can be effective for achieving a limited number of reprocessing cycles.^26^^,^^27^^,^^37^^,^^38^ This approach exploits the ability of UVC light from 200-260 nm to damage DNA.^38^

While mercury vapour lamps can be used as a source of UVC light, there is now growing interest in using LED light sources which emit at 222 nm, due to their greater efficiency and longer life. Further work is needed to determine how many exposures to UVC radiation a mask or respirator can undergo before degradation of the polymer layers occur.

Existing work using 254 nm UVC light has employed relatively long exposure times (eg, 3 hours), but these can be reduced to more workable times such as 15 or 30 minutes by increasing the power density of the UVC light being delivered to more than 20 Joules per square centimetre.^27^

### Various solvents and solutions

A concern is that a potential reprocessing treatment might reduce the electrostatic charge present on the polypropylene fibres produced using melt-blown extrusion that form the so-called electret filter layer. This charge plays an important role in attracting and retaining small particles, especially in the submicron range. The problem of a loss of charge leading to impaired filtration performance has been seen when masks and respirators have been washed with soap and water, sprayed with isopropanol, soaked in sodium hypochlorite, or soaked in ethanol or isopropanol.^20^ As well, organic solvents, including acetone and toluene, can also increase charge mobility in polypropylene fibres, leading to a loss of charge and thus a drop in submicron particle filtration effectiveness.^41^^,^^42^

This phenomena of charge loss occurs also during clinical use, when the external surface of a mask or respirator becomes contaminated by splashes of fluids, and the inner surface is exposed to exhaled moisture, which then condenses onto the fibres. When fluid is present this leads to the flow of electrons, which neutralises the charge on the fibres. This loss of charge problem sits behind various recommendations from manufacturers that the duration of use for surgical masks and surgical respirators should not exceed 4 hours and 8 hours respectively.^21^ It is possible that including a moisture absorbing agent (such as a super absorbent polymer) could extend the use time by absorbing moisture that is exhaled by the user, and at the same time improving their comfort.^39^

## Alternative mask materials

Bioplastics are a potential alternative material for the fabrication of PPE. These plastics are produced from renewable resources (such as sugarcane). Bioplastics are not a panacea for the issues around PPE. When considering such natural sources, one must factor in competition with agriculture for food production, and also account for water usage. Any new materials in this space need to be tested thoroughly in terms of their biodegradability or their ability to be recycled.^1^

Replacing polypropylene with cotton or cellulose based nonwoven fabrics is a potential strategy to reduce the environmental impact of masks, respirators, and gowns. These natural materials are hydrophilic and therefore would need to be coated with a suitable polymer to make the outer surface fluid resistant. The mask would still follow the traditional multilayered design, and the middle layer could also incorporate antimicrobial agents that have been covalently bonded, to prevent them leaching over time when the mask is washed.^40^

Another material of interest is biopolymers derived from the wheat gluten produced during the manufacture of cereals. This material can be electrospun into nanofibres and used to create gluten-based facemasks.^42^ Other potential biomaterials of interest include cellulose acetate, alginates, chitins and chitosan.^43^^,^^44^

With alternative materials of natural origin, extended use and episodes of re-use will lead to degradation, and declining performance for filtration over time. Manufacturers of such masks for consumer use specify maximum values for cycles of re-use, such as 30 for cotton masks, and 25 for bamboo masks, versus 50 for polyester masks made from synthetic fibres. While polyester masks have a longer service life, washing these generates microplastics and microfibres, both of which are problematic when discharged into water treatment systems.^45^ Part of safe laundering involves using a lint filter to collect microfibres that are shared from the laundering process.^45^

When considering reusable masks, attention must be paid to the laundry protocol, specifically the use of machine laundering rather than laundering by hand. The latter is more likely to lead to infectious material remaining on the mask, allowing it to serve as a fomite, while the former will render the mask safe for reuse, especially if a bleach additive is included with the wash.^6^

It remains to be seen how far masks made of natural origin can be used in a healthcare setting such as a dental clinic. A range of options can be considered, including masks made completely of natural materials, as well as those made from recycled or regenerated materials, and masks which are a combination of different technologies, such as washable bamboo for the inner and outer parts, with replaceable segments that contain the nano filters for submicron filtration. Such combinations can provide the benefits of natural fibre materials and washability, and greatly reduce the need for polypropylene fibres. A key consideration will be that the fibres of the main parts of the mask are biodegradable or able to be reused, or both.

Whatever approach is taken, it is essential that clear protocols are developed for the cleaning and decontamination of any items of PPE that are to be reused. When such protocols are in place and are used routinely, items of PPE that are reused can provide protection but not pose risks for transmission of infection to staff.^46^

Attempts have been made to fabricate masks from fabric sterilisation wrapping materials, such as one or more layers of Halyard H300, H500 or H600 polypropylene wrap, on the basis that these materials were originally designed to provide a sterile barrier against pathogens to protect wrapped items. Masks made from such materials can withstand steam sterilisation up to 5 times and still have sufficient filtration performance and splash resistance.^47^ The more layers of material that are used, the greater the resistance to the movement of air. A challenge with using H500 and H600, which are designed for wrapping heavier items for sterilisation, is that when used for filtration purposes they give a higher breathing resistance than a conventional surgical mask. Various designs for fabricating respirators using such materials have been published.^47^

## Designed-for-purpose reusable masks and respirators

### Technology examples

Due to recent developments in technology, masks and respirators designed for many cycles of reuse are starting to appear in various parts of the world. Two examples will now be discussed. The first of these is the Sonovia mask from Israel. This has zinc oxide and copper oxide nanoparticles incorporated into the fibres using a sonochemical process. These particles allow the mask to retain useful antibacterial activity for its stated life cycle of 65 wash cycles in hot water (75 °C), whilst maintaining suitable mask performance for filtration (98% bacterial filtration efficiency).^48^^,^^49^

The second example is Etrema from Canada, who developed a range of surgical masks and surgical respirators. These have high breathability (below 50% of the allowed resistance to inhalation and exhalation) and maintain over 95% submicron particle filtration efficiency after 100 washes. These masks and respirators gained registration with Health Canada (licence 22723) for use in healthcare settings. These are both excellent examples of technology developments that can considerably lower the numbers of masks of respirators that dental staff use.

### Avoiding charge degradation

There are several ways that masks designed for repeated reprocessing of such products can avoid the problems of irreversible deterioration in submicron particle filtration caused by loss of charge. The first of these is by relying on mechanical filtration rather than charge to achieve the required filtration efficiency. In this way, the mask or respirator will be able to sustain processes such as washing to remove contamination on both the outer and the inner surfaces.

A second approach is to include nanoscale generators within the mask or respirator, to cause recharging of the inner electret fibres. Such generators can be based on coatings of polyvinylidene fluoride and graphene oxide which generate the tribo-electric voltage that recharges the polypropylene fibres. Such nanogenerators are powered by mechanical movements of the mask or respirator, including regular breathing as well as rubbing or tapping the surface.^40^

### Environmental impacts of changing to reuseable masks

If a clinic were to adopt reusable masks, the potential benefits could be impressive. In terms of waste generation, if a single disposable mask was used every day for one year by just 1000 people, that would generate 1 ton of waste. On the other hand, changing to reuseable masks would create more than 85% less waste, with 3.5 times less climate impact (measured in terms of carbon dioxide equivalents). The change would lower costs by 3.7 times, even when factoring in the initial outlay and the ongoing costs of washing (including electricity and detergent). In terms of the impact on climate change, there is equivalency between using 1 single-use mask per day and using 48 reusable masks, with each of these washed 30 times before replacement.^6^

A similar line of argument applies for half-face reuseable elastomeric respirators (eg, Draeger) with replaceable filter inserts. These respirators are designed to be cleaned after use, and the filter inserts have a life of several weeks.^50^^,^^51^ Such devices maintain a good seal against the face over extended periods of use, and are comfortable to wear.^51^ When re-used, it is important to note that information provided from the manufacturer for cleaning and disinfection may be insufficient for safe work practices, and thus specific standard operating procedures need to be developed. Without specific training or supervision, those who are attempting to clean and disinfect reusable elastomeric respirators are very likely to make major procedural errors, however this issue resolves when well-designed standard operating procedures have been developed and are closely adhered to.^50^

## Disposable versus reuseable gowns

Disposable polyester and polypropylene gowns have been available for more than 30 years, while the concept of using sterilised gowns for surgery dates to 1883.^52^ During the COVID-19 pandemic, demand for disposable gowns skyrocketed, resulting in shortages in both non-sterile disposable gowns as well as in sterile disposable gowns. In some settings where the use of imported disposable materials was not possible, healthcare facilities resorted to using locally produced washable woven cotton gowns. These were soaked in sodium hypochlorite before being washed at 60 °C.^53^ Normally, such gowns would be washed without exposure to sodium hypochlorite, and laundered and re-used 75 times before disposal.^8^

### Laundering of reuseable gowns

Modern reuseable (washable) cotton and cotton-polyester or polyester gowns with high thread counts are still used widely for surgical procedures in many parts of the world. A key point of difference between pure cotton gowns and gowns containing polyester is that the latter have a shorter drying time but need to be machine-dried at a lower temperature than gowns made of pure cotton. In a hospital setting, a typical laundry process for gowns involves washing in hot water (71 °C for 25 minutes), followed by drying (65-76 °C for 30 minutes). Both the washing and machine drying components have microbiocidal actions.^54^ Importantly, the laundering process has no detrimental effect on the water-resistance of reusable cloth gowns.^55^

Using reusable linen products for gowns can lead to worthwhile cost savings in consumable supplies for clinics, without concerns around performance, since modern reusable fabrics are at least equal to their disposable counterparts in terms of being fluid repellent.^17^^,^^56^ Some studies have shown that reusable gowns are superior in performance to disposable gowns, and offer increased protection from fluid splashes and significant cost savings to healthcare facilities,^55^ while other studies have shown that most people (80% or more) feel comfortable and safe wearing reusable gowns, and have confidence in the cleaning process. Ratings for breathability of the gowns are lower, at around 45%.^57^ Further improvements in fabrics used in gowns may address this issue.

One must consider the labour costs for laundry, the impact on water use, and the generation of grey water containing detergents. These need to be weighed against the inherent environmental costs for landfill or incineration for disposable gowns. While healthcare facilities may not have been a major contributor to the total amount of refuse deposited into landfills prior to the pandemic,^17^ that situation changed with the extensive use of disposable gowns during the COVID-19 pandemic as a universally accepted method of reducing risk to staff.

### Environmental impacts of disposable gowns

In terms of greenhouse gas emissions by items of PPE, disposable gowns are a major contributor, being typically greater than disposable gloves, and many times higher than masks or respirators.^57^ The environmental impacts of reusable gowns are much lower than those for disposable gowns by considerable margins, in terms of greenhouse gas emissions (by 66%), energy consumption (by 64%), and the generation of solid waste (by 84%). Said another way, changing from disposable gowns to reusable gowns can reduce the carbon footprint of gowns by two-thirds.^58^

## Future directions for research

The question of degradation of masks, respirators and gowns by successive reprocessing cycles needs to be explored in detail, to inform the optimisation of existing processes using UVC, VHP and other methods, as well as to inform further developments of substrate materials, such as polymers that can withstand multiple exposures to these treatments. A range of antimicrobial and self-disinfecting characteristics have been developed for masks and respirators,^40^ including silver and copper-based nanoparticles and nanowires, and many of these will be able to withstand reprocessing using UVC or VHP.

Future research needs to consider how facemasks and respirators that are designed to be reprocessed influence human respiration and also the thermoregulation of facial skin beneath the mask. Masks and respirators with greater breathability will lower the demands for the user during inspiration and expiration, and reduce the development of an adverse microenvironment on the facial skin surface. Likewise, the materials used should not impede heat exchange between the facial skin and the surrounding environment.^59^ Moreover, future designs should also consider including some means to lower the moisture content within the mask or respirator, to not only improve wearer comfort, but also to prevent wicking across the mask or respirator caused by moisture that condenses during extended periods of wear.

Future investigations can build upon existing work using photocatalytic agents such as titanium dioxide nanoparticles or nanowires. These can be included to enhance the antimicrobial actions of sunlight or short wavelength ultraviolet light, by releasing reactive oxygen species.^40^ This can, in turn, inactivate contaminants on the inner and outer services of masks. Whenever such additional chemical agents are included, studies are needed to document how much the photosensitiser is washed away over repeated reprocessing cycles.

Similar issues apply to the concept of including immobilised graphene oxide, where polymerisation of a cross-linker can prevent the graphene oxide from being washed out quickly when the item is washed. Impregnating silver or copper nanoparticles into the fibres should reduce problems of loss and degradation over time.

For masks, respirators and gowns, past research indicates that user comfort is not adversely impacted by antimicrobial finishes on the surface,^59^ and hence such features should be included, to improve the protection offered to the wearer. A final area for improvements in PPE relates to ‘design for human variability’, so that one size of a particular item of PPE can fit a wider range of body shapes and sizes.

## Regulatory challenges

As discussed above, for certain forms of PPE, reuse can be a practical alternative. On the other hand, an ongoing emphasis on single use PPE is not sustainable for the future.^2^ Some governments have established policies that facilitate the use of reusable alternatives or materials that can be recycled, rather than insisting on single use PPE.^14^ There is no reason that the key sustainability principles of reduce, reuse, and recycle cannot be applied to PPE, to align with the United Nations sustainable development goals, including climate action, and responsible consumption and production.

One of the difficulties going forward for reusable PPE is the lack of standards that apply specifically to reusable PPE. The wording of some existing standards for PPE can be problematic in that they refer explicitly to single use PPE. Going forward, it will be necessary to either include reuseable PPE into revisions of existing standards for PPE, or to develop new standards explicitly for reusable forms of PPE. If the latter pathway is chosen, key criteria for performance can be adopted, including in the case of masks and respirators, providing adequate protection from particles at various stated levels of efficacy, and for masks respirators and gowns, resistance to blood splatter and other fluids. Any such new or revised standards will need to consider the extent of testing that manufacturers will be required to perform to establish claims of reuse, and the number of reuse cycles before disposal.

National and regional governments can provide business incentives to encourage manufacturers to voluntarily repurpose their production facilities to make reuseable PPE. During the pandemic, the manufacturing of masks and respirators diversified around the globe, and this was driven by incentives and opportunities, as well as by unmet local demands. Likewise, local manufacture of reusable forms of PPE should be encouraged. Local manufacturing reduces the likelihood of supply chain disruptions, and facilitates logistics and deliveries for clinics that are based in the same country.

Another practical issue which arises is that national infection control guidelines will need to be updated to take on board the existence of reusable forms of PPE. If this is not done, situations will arise where a reusable PPE item (eg, a surgical mask or respirator) is approved by the medical regulator for that country, but its use is not adopted by the dental clinics in that same country because their national protocols, which were written long before reusable PPE was developed, stipulate that particular PPE items must be single use and disposable. Dental boards and other regulators need to be on the front foot in incentivising practices to adopt appropriate forms of reusable PPE as this is not only economically efficient for the practice, but also reduce the impact on the environment of using PPE.

A final important area of work is to achieve closer alignment on methods used to test the performance of masks, respirators and gowns, for both disposable and reusable types. At the present time, standards and test procedures vary across the globe, and this leads to some confusion in terms of product labelling.^60^ The current performance tests are well understood, but may not align closely with challenges faced in clinical use.

## Conclusions

Decisions about items of PPE extend well beyond containing direct costs and ensuring compliance with relevant standards and performance expectations. It is important that end users consider indirect costs and impacts of PPE on the environment. Such impacts occur from manufacture, transport, and disposal of PPE, and from degradation within the environment. Those making decisions on product use need to consider all these elements, and scrutinise the alternatives, going well beyond the issue of direct costs.

There are strong marketing campaigns around biodegradable items, and those charged with making purchasing decisions need to be very careful of attempts at ‘greenwashing’, where certain products are claimed to have a reduced environmental impact compared with competitor products on the market. The current reality is that single use PPE materials made almost entirely of polypropylene are neither recyclable nor readily degradable.^15^

When problems arise in the supply chain, the impacts of any disruption may be less where items are completely reusable, since the clinic requires less volume of PPE products to support its normal operations. Making a change to use more reusable items can be challenging when staff have a developed ‘single use throwaway’ mindset that ignores the issues of sustainability. While many have practiced in an era of ‘everything disposable’ for PPE, that phase arguably peaked during the COVID-19 pandemic and has now declined.

Going forward, increased research efforts are needed around the decontamination and reuse of PPE. As mentioned earlier, the platform that has been established by past research must be extended to consider in more detail the extent of degradation during each reprocessing cycle, and how to reliably estimate the point at which the item no longer meets its requirement to prevent the penetration of pathogens to the user.

At the same time, regulators in the infection control space need to pay more attention to sustainability issues and opportunities for reducing, reusing, and recycling that present as technology progresses forward, and align their requirements with the changing evidence base over time. Pandemics will occur again, so the search for sustainable solutions must remain a priority.

## Conflict of interest

The author has no conflicts of interest to declare.
